# Clinical practice guideline recommendation summaries for pediatric oncology health care professionals: A qualitative study

**DOI:** 10.1371/journal.pone.0281890

**Published:** 2023-02-21

**Authors:** Nancy Santesso, Melissa Beauchemin, Paula D. Robinson, Alexandra M. Walsh, Aaron J. Sugalski, Tammy Lo, Ha Dang, Brian T. Fisher, Allison C. Grimes, Andrea Rothfus Wrightson, Lolie C. Yu, Lillian Sung, L. Lee Dupuis

**Affiliations:** 1 Department of Health Research Methods, Evidence and Impact, McMaster University, Hamilton, Ontario, Canada; 2 Columbia University School of Nursing/Herbert Irving Cancer Center, New York, New York, United States of America; 3 Pediatric Oncology Group of Ontario, Toronto, Ontario, Canada; 4 Center for Cancer and Blood Disorders, Phoenix Children’s Hospital, University of Arizona, Phoenix, Arizona, United States of America; 5 University of Texas Health Science Center San Antonio, San Antonio, Texas, United States of America; 6 Department of Research and Evaluation, Kaiser Permanente, Pasadena, California, United States of America; 7 Biostatistics and Data Management, Johnson and Johnson Medical Devices Companies, Irvine, California, United States of America; 8 The Children’s Hospital of Philadelphia and the Perelman School of Medicine at the University of Pennsylvania, Philadelphia, Pennsylvania, United States of America; 9 Nemours Center for Cancer and Blood Disorders, Wilmington, Delaware, United States of America; 10 LSUHSC/Children’s Hospital, New Orleans, Louisiana, United States of America; 11 Division of Haematology/Oncology, The Hospital for Sick Children and Department of Paediatrics, University of Toronto, Toronto, Ontario, Canada; 12 Research Institute, The Hospital for Sick Children and Leslie Dan Faculty of Pharmacy, University of Toronto, Toronto, Ontario, Canada; Beatrix Children’s Hospital, University Medical Center Groningen, NETHERLANDS

## Abstract

**Objective:**

To develop a summary format of clinical practice guideline (CPG) recommendations to improve understandability among health care professionals.

**Methods:**

We developed a summary format based on current research and used the “Think Aloud” technique in one-on-one cognitive interviews to iteratively improve it. Interviews of health care professionals from Children’s Oncology Group-member, National Cancer Institute Community Oncology Research Program sites were conducted. After every five interviews (a round), responses were reviewed, and changes made to the format until it was well understood and no new, substantive suggestions for revision were raised. We took a directed (deductive) approach to content analysis of the interview notes to identify concerns related to recommendation summary usability, understandability, validity, applicability and visual appeal.

**Results:**

During seven rounds of interviews with 33 health care professionals, we identified important factors that influenced understandability. Participants found understanding weak recommendations more challenging than strong recommendations. Understanding was improved when the term ‘conditional’ recommendation was used instead of ‘weak’ recommendation. Participants found a Rationale section to be very helpful but desired more information when a recommendation entailed a practice change. In the final format, the recommendation strength is clearly indicated in the title, highlighted, and defined within a text box. The rationale for the recommendation is in a column on the left, with supporting evidence on the right. In a bulleted list, the Rationale section describes the benefits and harms and additional factors, such as implementation, that were considered by the CPG developers. Each bullet under the supporting evidence section indicates the level of evidence with an explanation and the supporting studies with hyperlinks when applicable.

**Conclusions:**

A summary format to present strong and conditional recommendations was created through an iterative interview process. The format is straightforward, making it easy for organizations and CPG developers to use it to communicate recommendations clearly to intended users.

## Introduction

Many organizations dedicate time and resources to develop clinical practice guidelines (CPGs). Guideline development groups spend countless hours crafting the recommendations and supporting materials, with the goal of improving uptake and implementation. Despite these efforts, implementation has proved challenging across many professions and disciplines. While much of the literature has focused on how to implement recommendations in practice (such as through auditing and providing feedback to practitioners), there has been increasing interest in determining the inherent features of the recommendations themselves and their format that could influence implementation [1, 2]. The guideline implementability framework of Gagliardi et al., for example, outlines four specific domains related to the presentation of recommendations that affect implementability: Usability, Applicability, Validity and Adaptability [3]. Usability refers to how the recommendation and the evidence is presented and can include how users navigate the information; applicability addresses the inclusion of contextual information to promote use of the recommendation for an individual; validity covers how the evidence is summarized and presented for ease of interpretation; and adaptability focuses on the different versions of a guideline to improve uptake. This framework has been incorporated into several recent implementability tools including the GUIDE-M, specifically in the ‘communicating content’ tactic [4].

With respect to usability, the layout and choice of wording of the recommendation, as well as the supporting evidence, could influence how well the recommendation’s message is textually communicated. Some guidance exists to improve the communication of the intended action of the recommendation. The National Academy of Science’s (formerly known as the Institute of Medicine) Standards for Developing Trustworthy Clinical Practice Guidelines indicate that recommendations should clearly describe the action and the strength of the recommendation: whether the recommendation should be in the affirmative (i.e. ‘to do something’) or the negative (i.e. ‘not do something’) and how to frame the level of obligation to follow the recommendation [5]. When using the Grading of Recommendation, Assessment, Development, and Evaluation (GRADE) approach, the strength of a recommendation is classified as either strong or weak/conditional [6]. Strong recommendations are associated with a high level of obligation to provide (or withhold) an intervention as the standard of care. Conversely, weak/conditional recommendations prompt clinicians to also consider values, preferences or other factors when applying the recommendation.

Based on these guidance statements for recommendation development, the wording of any recommendation should convey its strength intrinsically. However, the choice of word or wording to convey this strength has proven to be challenging. Investigators have evaluated ‘recommend’ versus ‘suggest’ and ‘should’ versus ‘might’, and found that no word or wording was superior in communicating the recommendation strength [7]. Others have examined wording such as “should”, “may” and “must” and found that ‘must‘ conveyed the highest level of obligation, and “may consider” the lowest level of obligation [8]. However, there was considerable overlap in the level of obligation conveyed.

It is also unclear how much information should accompany recommendations to ensure understanding and acceptance. Some research supports the need for more explanation when a recommendation is based on a close balance of the evidence, or when its implementation requires that other factors be considered [9]. These findings suggest that more rationale and implementation information is necessary for weak/conditional recommendations to be understood as compared to strong recommendations.

We undertook this qualitative study to develop a summary format to communicate CPG recommendations [10] to health care professionals, using examples in pediatric oncology. This multi-center, qualitative study is one of three sub-studies within a Children’s Oncology Group (COG) study (ACCL15N1CD). The overall goal is to investigate the use of CPGs at pediatric National Cancer Institute (NCI) Community Oncology Research Program (NCORP) sites by understanding current practice in supportive care, exploring the barriers and facilitators to CPG uptake and, finally, developing a format for recommendation summaries to improve their understandability.

## Methods

We conducted one-on-one interviews to develop a CPG recommendation summary format that is well understood. We used the “Think Aloud” (TAL) technique of cognitive interviewing in multiple rounds (groups) to iteratively improve the format by exploring participants’ understanding of the recommendation summaries and preferences for the presentation. We applied a directed content analysis to explore multiple themes from within the interviews. We used the Standards for Reporting Qualitative Research [11] to guide the report of our findings.

### Participants

All 37 COG-member NCORP sites were invited to participate in the primary study. We invited health care professionals who provided direct care for children with cancer at participating sites to be interviewed. Trainees were excluded. Potential participants submitted their demographic characteristics and were purposively sampled to ensure variability by type of health care professional, years in pediatric oncology practice, self-professed level of familiarity with CPGs (from not very to very aware), NCORP site location, and proportion of time devoted to direct patient care.

Ethics approval for the study was obtained from the National Cancer Institute Pediatric Central Institutional Review Board (OMB# 0925–0753). Participants gave verbal consent to be interviewed; the need for documentation of consent was waived.

### Development of the initial recommendation summary format

The initial recommendation summary format (S1 File) was developed over several virtual meetings by the study team (CPG developers, CPG methodologists and CPG users) with assistance from a graphic designer and informed by literature and health literacy principles. We built on previous work completed by the GRADE Working Group through the DECIDE project (www.decide-collaboration.eu) and on formats for different audiences freely available in the GRADEpro online software (www.gradepro.org). We considered the quantity of text (e.g., brief versus more detailed), the type of information (e.g., rationale and summary of evidence), formatting (e.g., use of bulleted lists, font size, typeface, text boxes), use of symbols, and use of color. From recommendations available from COG-endorsed supportive care CPGs [12], we chose three topics (fever and neutropenia; chemotherapy-induced nausea and vomiting; and platelet transfusions), and chose a weak/conditional and a strong recommendation from each. Topics and recommendations were chosen based on applicability across multiple health care institutions and health care professions. A total of six examples were drafted. Material (e.g. rationale, evidence) from the source CPG publications were used to populate the examples.

### Interviews

We conducted one-on-one, semi-structured interviews via video chat (utilizing Go-to-Meeting^™^) lasting approximately 30 to 60 minutes. Five members of the team conducted interviews (MB, LLD, PDR, AMW, NS) who had experience in health research methodology and guideline development, and/or paediatric oncology. Each session was recorded, and interviewers took notes during and after the interview—all without identifying the interviewee.

Each participant was randomly assigned a topic (fever and neutropenia; chemotherapy-induced nausea and vomiting; or platelet transfusions) and shown two recommendation summaries on that topic (1 weak/conditional and 1 strong) in random order. The formats were provided via email to each participant as a PDF document which could be viewed on screen or printed. At the beginning of the interview, we described a short clinical scenario related to the recommendation topic to place the following recommendations in context. Using the TAL technique, we asked each participant for their initial reactions to the recommendation summary, and then continually prompted the participant to think aloud while he/she interpreted each section of the recommendation summary (e.g. title, strength of recommendation and definition, recommendation, rationale, supporting evidence). To evaluate understanding, we asked the participant to choose from four possible courses of action suggested by the recommendation summary: to provide care (or not) as per the recommendation (strong recommendations) or likely provide care (or not) as per the recommendation (weak/ conditional recommendations). Next, the interviewer disclosed the correct course of action according to the interview guide and asked the participant to reflect upon which parts of the recommendation summary enhanced or detracted from the correct understanding, and to suggest modifications to improve understanding. The interview guide is available in S2 File.

The interviews were conducted in rounds of five, but each round could be stopped prior to completion of five interviews if feedback indicated that immediate revision to the recommendation summary format was needed. Interviews were conducted until the tested recommendation summary format was well understood (defined as correctly choosing the action recommended) by at least four of five interviewees, and until saturation of ideas (defined as receipt of no new, substantive suggestions) was achieved. We planned to interview 25 participants at minimum and estimated that up to 50 interviews could be required.

### Data analysis

Feedback about the format was categorised as positive (not requiring a modification), obstructive (preventing use of the recommendation), major (interviewee able to solve independently), minor (cosmetic), and suggestions for modification/improvement. In addition, we calculated the number of participants per round who correctly interpreted the course of action for the recommendation summary. The team met virtually after each round of interviews to review the interview notes and agreed by consensus about which revisions should be made. The recommendation summary format was then revised prior to the next round of interviews. In order to identify other concerns related to recommendation summary understandability, two investigators (NS and LLD) conducted a directed content analysis of the notes taken during and after each interview based on the implementability framework [3]. To ensure trustworthiness and credibility, the analysis was reviewed and revised by the other members of the interviewing team (MB, PDR, AMW).

## Results

Twenty-six of the 37 NCORP sites (70%) were involved in the primary study and participants were selected from 15 of these sites. Five investigators conducted a total of 33 interviews in seven rounds from February 1, 2019 through September 3, 2020. Participants included physicians, nurses, pharmacists and other health care professionals. Almost all reported that they were fairly or very aware of CPGs. Demographic characteristics of participants and their institutions are provided in Table 1.

**Table 1:** Characteristics of participants and their institutions.

Characteristic	Value (N = 33)
**Participant Characteristics**	
Female Sex, n (%)	27 (82%)
Profession, n (%)	
Physician	11 (33%)
Nurse	11 (33%)
Nurse Practitioner	4 (12%)
Pharmacist	3 (9%)
Other^a^	4 (12%)
Median years since completion of most recent health care professional training (IQR)	13 (6.5–22.5)
Median years of pediatric oncology experience (IQR)	13 (6.0–24.5)
Median years at current institution (IQR)	11.4 (3–17)
Median percentage of time spent providing direct patient care (IQR)	80 (50–95)
Self-assessed awareness of CPGs, n (%)	
Very Aware	9 (27%)
Fairly Aware	21 (64%)
Not Very Aware	2 (6%)
Missing	1 (3%)
**Site Characteristics**	
Site Location, n (%)	
Western US	9 (27%)
Southwestern US	7 (21%)
Northeastern US	5 (15%)
Southeastern US	6 (18%)
Midwestern US	6 (18%)
Site Type, n (%)	
Minority/Underserved Community (vs. Other)	15 (45%)
Pediatric (vs. Mixed Adult and Pediatric)	20 (61%)
Private (vs. Academic)	13 (39%)

^a^Child life specialist, physical therapist, psychologist

Abbreviations: IQR—interquartile range; CPG—clinical practice guideline; US—United States

**Fig 1:**
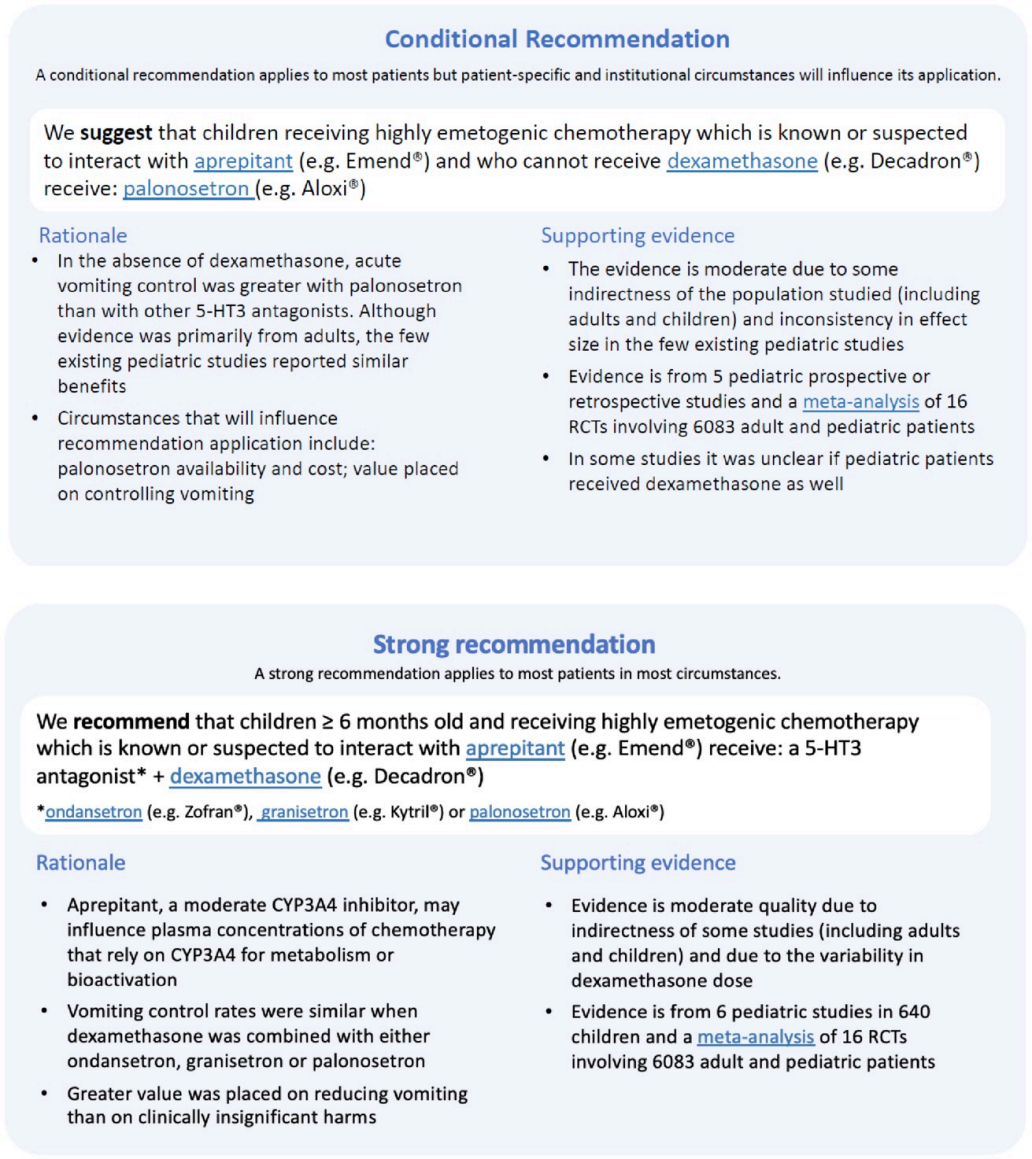
Final recommendation summary format: Chemotherapy-induced nausea and vomiting. Factors influencing understanding of the recommendation summaries.

Seven rounds of interviews were conducted in total. The first round was closed after three interviews because feedback on the initial format required immediate revision (e.g., changing paragraphs of text to bulleted lists). Overall, we found that major changes were made to the format for both the strong and weak/conditional recommendation summaries up to round 3 but there were few changes made in the subsequent rounds. In addition, during the first three rounds, we asked about understanding of the format based on a clinical scenario for an individual patient, but in subsequent rounds, we asked participants to respond from the perspective of their institution to encourage the participant to focus on the general application of the recommendation summary. By the sixth round of interviews, the strong recommendation summary format met criteria for both understanding and saturation. Therefore, only the weak/conditional format was discussed in the seventh round of interviews. Understanding of the recommendation summaries by participants in each round of interviews is presented in Table 2. Examples of the final recommendation formats are presented in Fig 1 for the topic of chemotherapy-induced nausea and vomiting.

**Table 2:** Number of participants with good understanding of the recommendation.

	Round 1	Round 2	Round 3	Round 4	Round 5	Round 6	Round 7
Weak/Conditional recommendation summary	3/3	2/5	2/5	5/5	2/5	1/5	5/5
Strong recommendation summary	1/3	5/5	4/5	4/5	5/5	5/5	*

* Strong recommendation summary format was not tested in Round 7.

### Usability

Throughout the interview a tension was noted between providing too much or too little information. During the first rounds when most of the information was presented in paragraphs of text, it became clear that interviewees found the provided information difficult to read. One participant stated, “because it’s too wordy I wouldn’t know where to start and would have to read every line.” Therefore, the format was changed to bulleted lists. Conversely, many participants wanted more and specific information about drugs that were recommended, such as brand and generic names, contraindications, and dosages. To address these conflicting requests, hyperlinks to additional information were embedded within the recommendation summaries. However, additional information provided in the Rationale section was well-liked and was, therefore retained: “I like the rationale—like in general—physicians like to see that rationale.” The positioning of the rationale on the left and then more details about the evidence on the right also made sense to interviewees.

In the early rounds of interviews, interviewees commented on the specific format of the recommendation summaries. Most comments were related to emphasizing key elements in the text, such as bolding the strength of the recommendation and the words used to denote the strength (e.g., ‘suggest’ or ‘use’), and changing the title of the document to include the strength of the recommendation. Participants also stated that strong and weak/conditional recommendation summaries should be presented in the same general format—a ‘consistent’ presentation.

### Understanding the strength of recommendations

The recommendations were first described in the formats as *strong* or *weak*. Strong recommendations are generally understood as meaning that the recommendation should be followed and were correctly interpreted by the majority of participants even in the early rounds of interviews (Table 2). The correct interpretation of the weak recommendations, however, was less clear to participants. The word *weak* was interpreted as “no better than a random recommendation” or “a failure” or “less important”. Participants reported that they would be less likely to follow a weak recommendation.

In consecutive rounds of interviews, we employed multiple strategies to communicate the meaning of a weak recommendation more clearly. First, we included a general statement describing the *weak* recommendation as “A **weak recommendation** will apply to the majority of patients, but may depend on circumstances, or patient or society values.” Unfortunately, this statement did not improve the understandability of the weak recommendation. Next, different symbols—a filled circle for strong, and a faded circle for weak—were used to distinguish strong from weak. Most participants indicated that the symbols simply looked like bullet points or were confusing or unnecessary and they were therefore eliminated. Then, different words were tried to convey the obligation to follow the recommendation. For example, strong recommendations had been written as ‘*we recommend’* using active verbs, such as *use* and had been well understood. Weak recommendations were often written as ‘*we suggest’* but interviewees were still confused about the intention of the recommendation [6, 7]. Finally, after six rounds of testing, we changed the word *weak* to *conditional* within the recommendation summaries and revised the general statements describing strong and conditional recommendations. At that point, participants correctly interpreted a conditional recommendation and appropriately conveyed the intent: “conditional just sounds more legitimate, and the word *conditional* is great as it is very neutral. You can use it for positive or negative.” Participants also remarked that *conditional* did not sound “inferior” and conveys, as is intended, the need for more thought when making decisions.

### Validity-Need for justification and rationale

The initial format of the recommendation summary included a brief Rationale section, where we summarized both the evidence and the justification for the recommendation from the original published CPG. Most participants indicated that the Rationale Section was very useful and helpful to understand why the recommendation was made. Participants appreciated the information provided about the number of studies or participants in the studies, the results, and the limitations of the evidence, as it showed that the recommendation was evidence-based. This section also included an overall rating of the evidence according to the GRADE approach, which was well-liked.

At times, however, participants had additional questions related to the evidence which was not provided within the Rationale section. Participants questioned why a weak/conditional recommendation was based on high quality evidence, or why a strong recommendation was based on moderate quality evidence. This juxtaposition was confusing. Some participants were also confused when the evidence was available but described as low quality. One participant stated “that is the part that doesn’t make sense to me. It is stating that it is weak and low quality but there is still a good amount of information… that is accurate information.” Others did not understand how evidence from research in adults could still be high quality when applied to children: “It’s hard because the data isn’t based on children so how can it be strong? But the evidence quality is high.” This suggested that more justification was needed to explain why evidence was rated at a specific quality level (e.g., because of limitations or risk of bias of the included studies, or the indirectness or applicability of the evidence).

Participants also suggested that the rationale could be communicated to a patient/patient’s family to explain why a medication or approach was being offered. In addition, they indicated that this section would be more useful if it described how institutional differences have or could have an impact on the recommendation. Since participants identified multiple roles for the Rationale section, it was divided into two sections in the third round to better meet these needs: Supporting Evidence and Rationale. The Rationale section describes the balance of benefits, and then harms, and provides information about additional factors, such as implementation, that were considered by the CPG panel when making the recommendation. The Supporting Evidence section explains the level of evidence with hyperlinks to supporting studies when applicable, and the quality of the evidence. Key words are hyperlinked to explanatory or supplementary information.

### Applicability-Acceptance and individualisation of a recommendation in practice

Similar to comments about needing a rationale (in particular for weak/conditional recommendations), participants noted that they wanted more information when a recommendation entailed an institutional practice change. Some acknowledged that a recommendation would be more easily accepted if it matched current practice: “this recommendation is standard practice, so I would do it.” Other factors related to acceptance were trust in the group who developed or endorsed the CPG and the level of supporting evidence. Even though the level of evidence is not correlated with the strength of the recommendation (e.g., a strong recommendation can be based on very low certainty evidence when there is the potential for serious harms), participants indicated that they would find it harder to accept a recommendation if it were based on low or very low-quality evidence. In addition, a participant stated that “…if it supports what I’m already doing, it doesn’t matter so much what the level of evidence is.”

To improve implementation of a recommendation, many participants requested more details, including information about the specific population to which the recommendation pertains and the circumstances. Suggestions included clearly indicating the population to which the recommendation applied in the recommendation statement and in the rationale; providing links to websites with additional descriptions or information, such as about drug doses; and providing brand names of the drugs mentioned. These changes were made after each round and improved understanding of the recommendation summary. Interviewees also expressed a desire for a care pathway or specific instructions, most notably when recommendations offered a choice (such as between one of several drugs). In later rounds, we changed the perspective by asking participants what the institutional standard of care should be based on the recommendation, i.e., using the recommendation to create care pathways which would apply to an individual patient. Still, there were some physician participants who indicated that they may not follow recommendations when deciding how to care for their own patients.

### Presentation of the recommendation summary-Final format

After six rounds, 28 interviews, the final format for a strong recommendation summary was reached whereas seven rounds and 33 interviews were required before the format for a conditional recommendation summary was finalised (Fig 1). These formats are presented as a single recommendation summary when printed or as single recommendation summaries with hyperlinks to additional information when presented electronically. The recommendation strength is clearly indicated in the title and, for clarity, the meaning of the strength of the recommendation follows just below. The recommendation is clearly stated and highlighted. The rationale is featured on the left side and the supporting evidence on the right side. Under each section, information that specifically addresses the findings that arose from the interviews is presented as a bulleted list.

## Discussion

Based on feedback from health care professionals in pediatric oncology, we created a summary format for the presentation of supportive care recommendations to improve recommendation usability, understandability, validity, applicability and visual appeal. In doing so, we obtained valuable insight into the preferences of users with respect to recommendation summary presentation and content as well as the challenges faced by users as they attempted to interpret the recommendations. We incorporated these findings into the final format. Recommendations in this summary format are intended to accompany a fully published CPG as an implementation tool.

One of the most striking issues that we uncovered was the misunderstanding of and discomfort with weak recommendations. This supports anecdotal evidence that practitioners may have difficulty understanding and applying recommendations that are weak, and that strong recommendations are more easily understood. Our final recommendation summary format successfully addressed this concern by including clear definitions of strong and weak recommendations, changing the word *weak* to *conditional*, providing a rationale for the recommendation, and including a summary of supporting evidence and factors for users to consider when implementing conditional recommendations. Despite the breadth of its content, the final recommendation summary format is brief.

We had also hypothesised that symbols might improve the understandability of recommendations, as they could be a way to distinguish the strength of the recommendation. We, however, did not find that the use of symbols or different shading had an impact on understanding and therefore removed symbols from the format early in the process.

We tested recommendations on three different supportive care topics with different levels of evidence associated with different strengths of recommendations. We were, therefore, able to explore participants’ understanding when the level of evidence did not intuitively match the strength of the recommendation—for example, a weak/conditional recommendation that was supported by moderate or high-quality evidence. These cases seemed the most confusing to the interviewees. However, by including some details about the evidence, (such as the limitation of the studies or the participants in the studies) interviewees understood the level of evidence and the strength of the recommendation. However, caution in describing the evidence is needed, as participants might use the description of the evidence to determine their own level of evidence, instead of considering the level assessed by the CPG developers.

An interesting finding of this study was the initial desire of many participants in early interviews for clinical pathways instead of recommendations. Clinical pathways are defined as tools that standardize care over a course of treatment by translating evidence into a structured, multidisciplinary care plan within a specific context [13]. A clinical pathway will include decisions regarding the use of available resources in a specific setting and thus will include information about the use of specific drugs and doses. A CPG may make a strong recommendation for the use of a drug class; a care pathway will state that a specific member of that drug class be used. Misunderstanding of the distinction between a care pathway and a CPG may explain why many participants desired specificity in the recommendation summary format. It may also explain why weak/conditional recommendations were less well understood, since a care pathway may offer little room for discretion even when based on a weak/conditional recommendation. It may also be for this reason that we found that most participants did not want more information presented in the recommendation summary format, but preferred links to other detailed information if needed. While multi-layered approaches—where increasing amounts of information are provided in layers—have been explored by other investigators [14], we did not perceive that participants wanted another layer of explanatory information as part of the format that specifically described the recommendation or the evidence. Nor did we find that more than a sentence about the implementation of the recommendation was needed. Nonetheless, if more information was desired, users could seek out the complete, published CPG.

One of the strengths of this study was our use of the TAL interview technique, which allowed us to glean valuable information about how people interpreted what we presented and consequently enabled us to modify the recommendation summary format to improve understanding. Further, inclusion of a broad range of health care professionals who provide direct care to pediatric oncology patients gave voice to users at all levels of the CPG implementation process: from those who select a CPG for implementation to those who deliver direct care. According to our protocol, the recommendation summary format was tested until it was well understood, defined as achieving saturation of ideas and having at least four out of five interviewees choose the correct action based on the recommendation. The former criterion is subjective. However, our research team of more than five members had to agree on this judgement, ensuring our results were reliable. Despite the many strengths of this study, there are limitations to consider. Our findings apply only to summaries of published CPG recommendations [10]. Expert opinion statements and good practice statements may well require a different format to promote understanding [15]. We interviewed health care professionals providing care only in the field of pediatric oncology, and it is unknown whether the results are directly applicable to other fields. As stated previously, pediatric cancer treatment is very protocolized and we might assume that participants would perceive recommendations as non-negotiable. However, we did not find this and therefore expect that the results of this study could apply directly to any health care professional regardless of whether protocols are commonly used. Our results are not generalizable to CPG recommendation summaries aimed at patients or their families. Although their perspectives are important to incorporate into CPG recommendations themselves, development of a CPG recommendation summary format that is well understood by patients and families will require a separate study. We also did not test whether the final recommendation summary format would lead to changes in practice, as we asked participants what they (or their institution) *would* do if given the recommendation summary. Some indicated that they may not provide CPG-consistent care. In future, we could evaluate changes in practice when CPGs using the new recommendation summary format are published are explore reasons for care choices that are not CPG-consistent. The format was also presented to participants in a package of materials, but not within a website or other electronic platform, such as an electronic order entry system. Although the recommendation summary format was tested on screen during most interviews, further exploration of the format within a platform could provide additional insight to its contribution to CPG-consistent care. We also focused only on a few key components of the implementability framework, but work continues to develop more tools to optimize communication of CPG content [4, 16].

## Conclusions

We provide a format to present a summary of strong and conditional recommendations that can be used to convey recommendations to health care professionals. The format is straightforward, making it easy for organizations and CPG developers to use it to summarize and communicate their recommendations clearly to intended users. Ideally, the implementability of this format could be assessed in a larger group of multi-disciplinary and multi-professional users. Once refined and integrated into CPGs, its influence on CPG-consistent care delivery should be assessed.

## Supporting information

S1 File Initial recommendation formats. (DOCX)

S2 File Interview guide. (DOCX)
