# Supplementary material for: Clinical practice guideline recommendation summaries for pediatric oncology health care professionals: A qualitative study
Source: PLoS One. 2023 Feb 21;18(2):e0281890. doi: 10.1371/journal.pone.0281890 (PMC9943009; doi:10.1371/journal.pone.0281890)

**S1 File. Initial Recommendation Formats**

**1.1 Chemotherapy-induced Nausea and Vomiting**

Weak recommendation
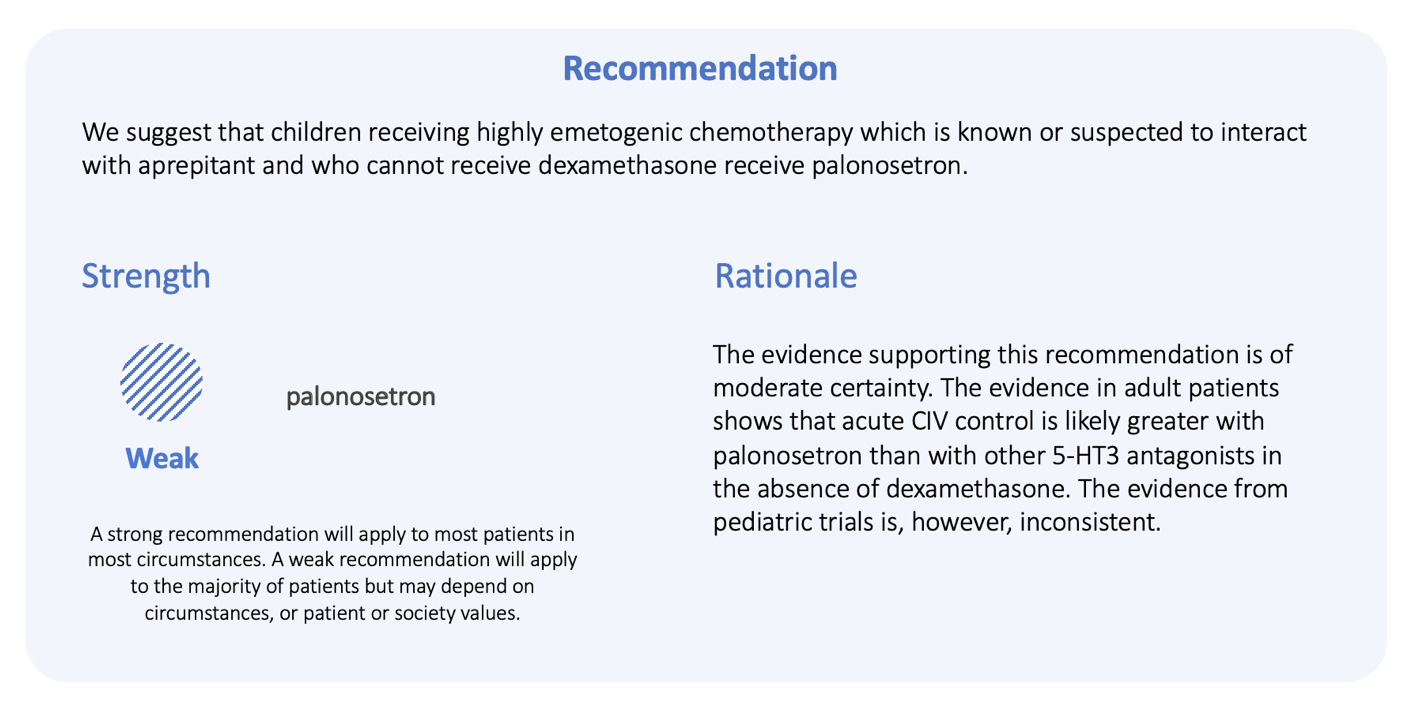


Strong recommendation
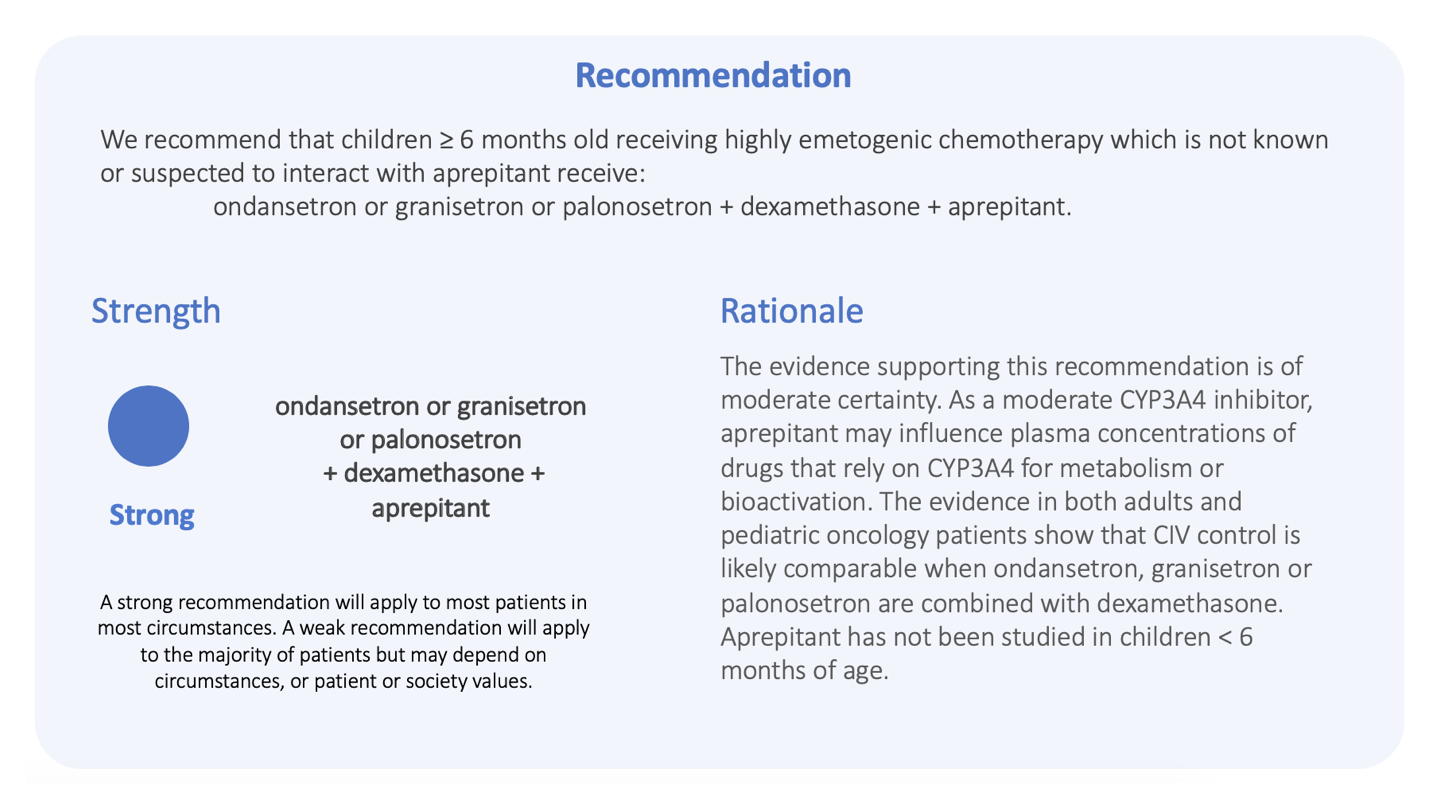


**1.2 Fever and Neutropenia**

Weak recommendation


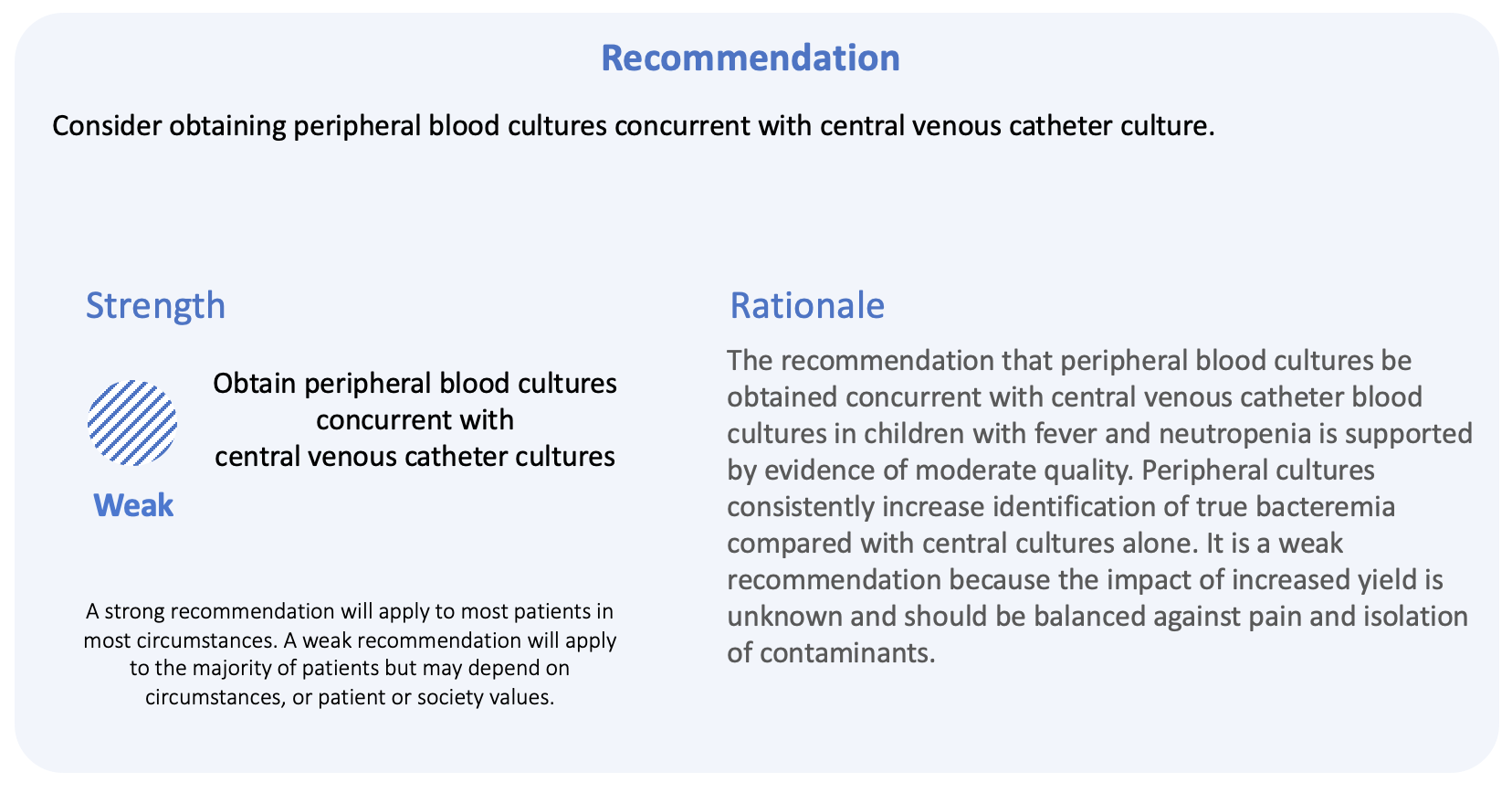


Strong recommendation


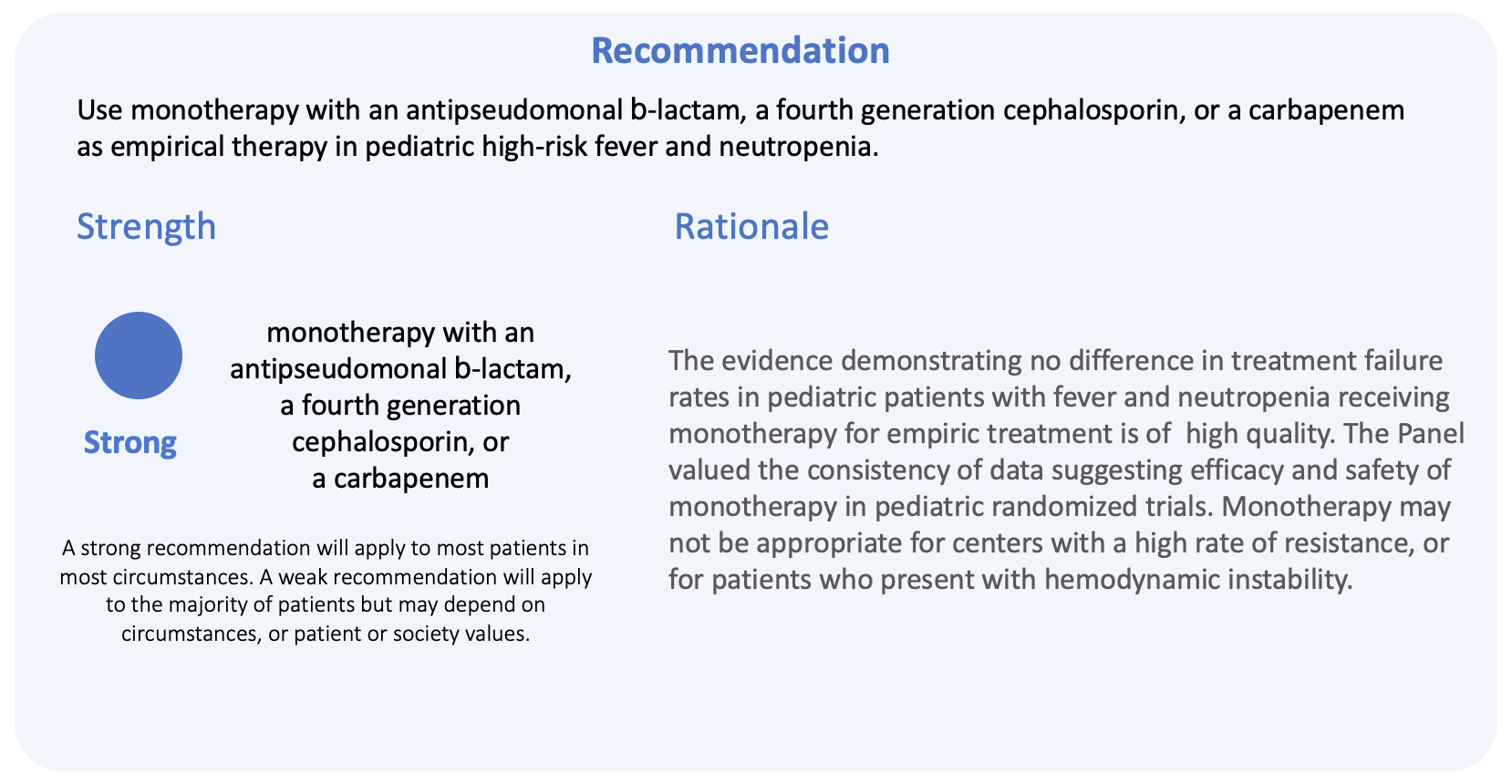


**1.3 Platelet Transfusion**

Weak recommendation


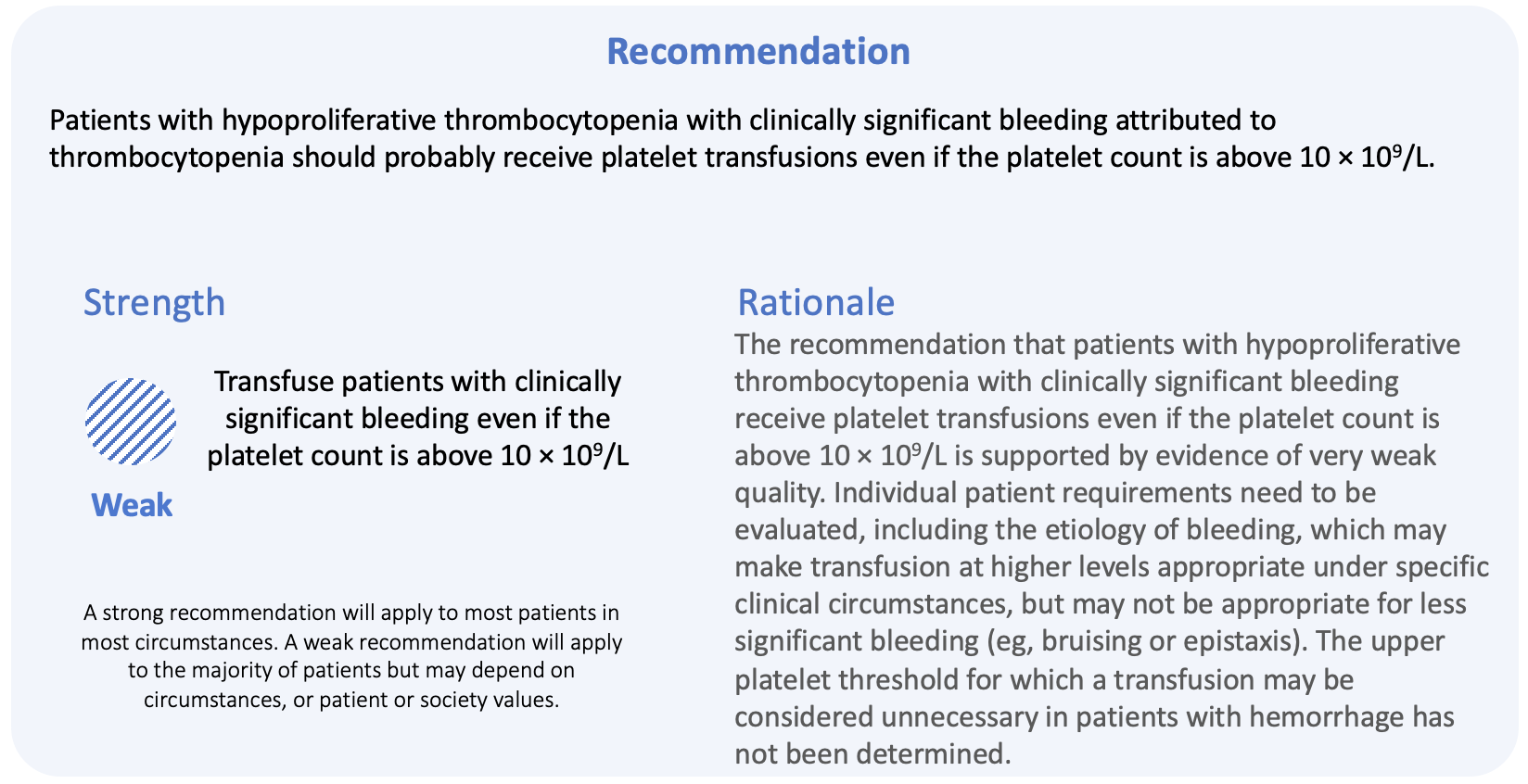


Strong recommendation


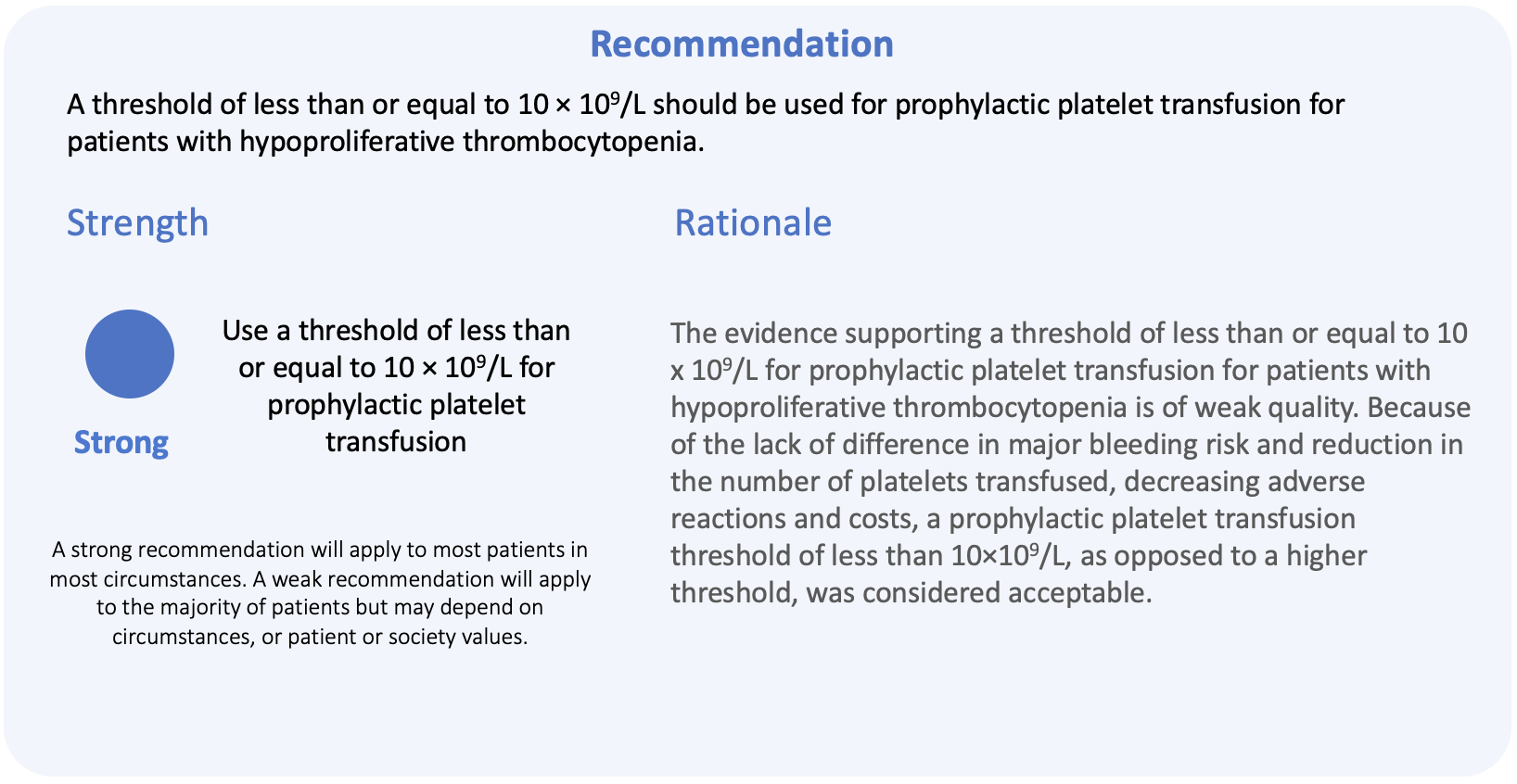

Supplement: S1 File — (DOCX) [file pone.0281890.s001.docx]
