# Supplementary material for: Clinical practice guideline recommendation summaries for pediatric oncology health care professionals: A qualitative study
Source: PLoS One. 2023 Feb 21;18(2):e0281890. doi: 10.1371/journal.pone.0281890 (PMC9943009; doi:10.1371/journal.pone.0281890)
Supplement: S2 File — (DOCX) [file pone.0281890.s002.docx]

**S2 File. Interview Guide**

**Participant Packages** delivered by Research Coordinator before interview (5 days ahead of time):

- - - - Full guideline
- Description of project – information sheet for participants with additional links to materials about the use of guidelines in the Children’s Oncology Group (COG)

Interviews to take place on **GOTOMEETING**

**Present:** Interviewer and participant

**Guide format**

*Italics are notes for you.*

Normal font is suggested wording of your dialogue.

# Interview Guide

*BEFORE STARTING THE INTERVIEW FORMALLY, REMIND THE PARTICIPANT THAT WE WILL BE RECORDING AND* ***START THE RECORDING NOW****.*

*Thank participant and introduce yourself, and ask participant to introduce himself/herself (i.e., to describe role at institution).*

As we indicated previously, we are interviewing health care professionals for feedback about the way we write clinical practice guidelines and recommendations. We will show you some examples of recommendations that are formatted in different ways and ask for your feedback.

The interview should take no more than an hour. We will be recording the interview and also taking notes. We will not use your name when we describe the results in papers – it is anonymous, and all your responses will be confidential.

We are testing out our material – so it is not about testing you. We want to know if the different formats to communicate a recommendation are useful. There are no right or wrong answers to our questions. If you think something is easy or difficult, clear or confusing, if you understand or don’t understand, we just want to know about it. We’d like you to think out loud during the interview: Tell me what you are thinking, what you see, what you find confusing or surprising, even the least little bit. For instance, if there are things you don’t understand, just say “I don’t know what this means...”. And remember we want your opinion not what you think others would think.

Any questions so far?

We have created different ways to present clinical practice guidelines to health care professionals. In your package, we included an example guideline called “Guideline” about the [insert guideline topic here]. You can open the document now or I am also showing it on the screen. Briefly, health care societies, such as the American Society of Clinical Oncology, gathered together a group of key stakeholders, reviewed the evidence for children with cancer, and developed recommendations to guide health care professionals about how to treat, manage or monitor their patients. Generally, the recommendations are written up in a standard document. We have some ideas about how to present the recommendations but would like your feedback.

Let’s start.

*Pick the scenario that applies to the recommendations the interviewee is randomized to discuss.*

*Chemotherapy-induced Nausea and Vomiting:*

Imagine that you are helping to decide what antiemetic prophylaxis will be given to patients at your hospital who receive highly emetogenic chemotherapy. You find the guidelines for the Prevention of Acute Nausea and Vomiting.

*Fever and Neutropenia:*

Imagine that you are helping to decide what investigations will be done at your hospital to identify the cause of fever in neutropenic pediatric patients and what antibiotics they will get. You find the guideline for the management of fever and neutropenia in children.

*Platelet Transfusion:*

Imagine that you are helping to decide what the thresholds for platelet transfusion for pediatric oncology patients will be at your hospital. You find the guidelines for platelet transfusion for cancer.

I am showing you a document on my screen. *Offer only if screen sharing not functioning:* Please open the document called ‘**Format 1/2**’. *Ensure that the order follows the assigned randomization.*

Please take a minute to read this recommendation. You don’t need to read it in too much detail now.

1. How interested are you in reading this recommendation?
2. What is your general impression of how it is written?

Now let’s talk about the specifics of the recommendation.

1. Let’s focus on the **wording** of this recommendation. If you had to make a decision about what to provide to patients at your hospital, would this recommendation be useful? Why or why not?

*Ask about whether the participant wants more or less information*

What would you choose for patients at your hospital based on this recommendation?

*In listening to the interviewee’s responses, indicate which of the options below they say they would take:*

a. *Provide care as per the recommendation [appropriate for strong recommendation]*

*b. Likely provide care as per the recommendation [appropriate for weak recommendation]*

*c. Likely not provide care as per the recommendation*

*d. Not provide care as per the recommendation*

1. Why did you decide to make this decision? Please walk me through your decision making process.
2. The developers of this recommendation intended for clinicians to:

*for STRONG recommendation:* provide care as per the recommendation

*for WEAK recommendation:* assess patient circumstances, likely provide care as per the recommendation to the majority of patients*”*.

*Insert language from the recommendation as appropriate in highlighted portion*

Which parts of the recommendation enhanced or detracted you from the intended message?

1. How could the recommendation be rewritten to improve your understanding of the intended message?

*a. Ask about the wording of the strength of the recommendation (strong versus weak; suggest versus recommend; use versus consider; should versus should probably);*

*b. Ask about the amount of information and type of information provided in the justification and implementation sections*

*c. Ask about the certainty of the evidence (in the justification) and how it is related to obligation to do the recommendation or strength of recommendation*

1. Now let’s focus on the **format** of the recommendation, in particular the figures and text. What do you like or dislike about the way the information is presented? Do you think that an icon to tell you that this is a weak/strong recommendation would be useful as a visual cue?
2. If you could change the format, what would you change?

*Ask about the following:*

- *sequence of information - length*
- *bolding - color*
- *spacing - pictures/icons/figures*

Now we will consider another recommendation format.

I am showing you a document on my screen. *Offer only if screen sharing not functioning:* Please open the document called ‘**Format 1/2**’**.** *Ensure that the order follows the assigned randomization.*

Please take a minute to read this recommendation. You don’t need to read it in too much detail now.

1. How interested are you in reading this recommendation?
2. What is your general impression of how it is written?

Now let’s talk about the specifics of the recommendation.

1. Let’s focus on the **wording** of this recommendation. If you had to make a decision about what to provide to your patient, would this recommendation be useful? Why or why not?

*Ask about whether the participant wants more or less information*

What would you choose for patients at your hospital based on this recommendation?

*In listening to the interviewee’s responses, indicate which of the options below they say they would take:*

a. *Provide care as per the recommendation [appropriate for strong recommendation]*

*b. Likely provide care as per the recommendation [appropriate for weak recommendation]*

*c. Likely not provide care as per the recommendation*

*d. Not provide care as per the recommendation*

1. Why did you decide to make this decision? Please walk me through your decision making process.
2. The developers of this recommendation intended for clinicians to:

*for STRONG recommendation:* provide care as per the recommendation

*for WEAK recommendation:* assess patient circumstances, likely provide care as per the recommendation to the majority of patients*”*.

*Insert language from the recommendation as appropriate in highlighted portion*

Which parts of the recommendation enhanced or detracted you from the intended message?

1. How could the recommendation be rewritten to improve your understanding of the intended message?

*a. Ask about the wording of the strength of the recommendation (strong versus weak; suggest or recommend; use versus consider; should versus should probably);*

*b. Ask about the amount of information and type of information provided in the justification and implementation sections*

*c. Ask about the certainty of the evidence (in the justification) and how it is related to obligation to do the recommendation or strength of recommendation*

1. Now let’s focus on the **format** of the recommendation, in particular the figures and text. What do you like or dislike about the way the information is presented? Do you think that an icon to tell you that this is a weak/strong recommendation would be useful as a visual cue?
2. If you could change the format, what would you change?

*Ask about the following:*

- - *sequence of information - length*
  - *bolding - color*
  - *spacing - pictures/icons/figures*

1. Thinking about your own practice, how would the content of this recommendation, help or hinder you from applying it to your practice?

*Ask as follow-up, if they would like a separate section for implementation tips. How helpful do you think it would be: not helpful to helpful*

Now we’d like to ask you about your **overall reflections** about the formats for a weak and strong recommendation

1. Do you think you need more or less information or different types of information depending on the strength of the recommended action? And what are your main reasons why?
2. For an individual recommendation, do you think that the overall format should be different depending on the strength of the recommended action? And what are your main reasons why?

Those are all the questions we wanted to cover, is there anything else that you’d like to mention about the formats or content of the recommendations before we finish?

Again, thank you for your time and participation. If you think of any additional thoughts over the next few weeks, please email me. We’re happy to receive any feedback.

Also, we will be analyzing the results over the next few months, and if we have any questions about the content of your interview, could we contact you again to clarify?
